# Prevalence of Surgery Among Individuals in the United States

**DOI:** 10.1097/AS9.0000000000000421

**Published:** 2024-04-11

**Authors:** Mark C. Bicket, Kao-Ping Chua, Pooja Lagisetty, Yi Li, Jennifer F. Waljee, Chad M. Brummett, Thuy D. Nguyen

**Affiliations:** From the * Department of Anesthesiology, University of Michigan Medical School; † Department of Pediatrics, University of Michigan Medical School; ‡ Department of Internal Medicine, University of Michigan Medical School; § Department of Biostatistics, University of Michigan School of Public Health; ‖ Department of Surgery, University of Michigan Medical School; ¶ Department of Anesthesiology, University of Michigan Medical School; # Department of Health Management and Policy, University of Michigan School of Public Health, Ann Arbor, MI.

## INTRODUCTION

Surgery represents an important event in the lives of many patients, their families, and communities. Surgical health care expenditures represent a rising cost, accounting for more than one-quarter of all healthcare-associated costs in the United States.^1,2^ Prior projections of surgical rates in the United States have ranged from 12.0 to 21.4 operations per 100,000 persons, though these volumes use data from 2007 or before.^3,4^ While more current estimates for outpatient surgeries have been described, much less is known regarding the recent prevalence of surgery in the United States across all care domains.^5^ Further, accurate estimates of the attributes of persons who undergo surgery, whether overall or by important characteristics such as age and sex, are not readily available. To address these gaps, we sought to analyze nationally representative data to understand the proportions of persons who undergo surgery every year.

## METHODS

This cross-sectional study investigated 2018 National Health Interview Survey (NHIS) data,^6,7^ which provides nationally representative estimates based on household interviews of the civilian noninstitutionalized population in the United States. The survey collects data from households using a stratified design with experienced interviewers and quality control measures to ensure robust participation and minimize biases. This study was exempted from review by the University of Michigan IRB and followed strengthening the reporting of observational studies in epidemiology reporting guidelines.

For the NHIS sample adult component, the conditional-response rate that measures the number of interviewed persons among those within households that completed a household roster was 83.9%, and the final response rate that measured both contacted and noncontacted eligible persons was 53.1%.^8^ For the NHIS sample pediatric component, the condition and final response rates were 93.5% and 59.2%, respectively. We examined the outcome of whether respondents reported having surgery in the past year based on the question, “During the past 12 months, (have you/has sample child) had surgery or other surgical procedures either as an inpatient or outpatient?” (yes/no). For the outcome, responses of “don’t know,” “not ascertained,” and “refused” were set to missing. Respondent characteristics included self-reported sex, age in years, race/ethnicity (categorized as Hispanic, non-Hispanic White, non-Hispanic Black, non-Hispanic Asian, and non-Hispanic other), and health insurance. Persons with multiple types of health insurance were assigned one category using the hierarchy: private, Medicare, Medicaid, other coverage, and uninsured, which included unknown.

Analyses incorporated survey sampling weights and strata to generate and enhance the representativeness of estimates by accounting for nonresponse and oversampling. Using the Stata -*svy*- command on complete cases, we examined descriptive statistics for the sample and estimated the proportion of persons who had at least one surgery in the past year overall and for subgroups based on respondent characteristics. Analyses used Stata software version 18 (StataCorp).

## RESULTS

The study population included 33,366 persons [mean age (standard error) 39.8 (0.2) years, 52.6% female], with 16.2% Hispanic, 62.7% non-Hispanic White, 12.3% non-Hispanic Black, 5.3% non-Hispanic Asian, and 3.5% non-Hispanic other race. Most reported having private health insurance (61.8%), with smaller proportions of Medicare (11.9%), Medicaid (13.3%), other insurance (4.1%), or being uninsured or having unknown insurance status (8.9%).

**Figure 1.**
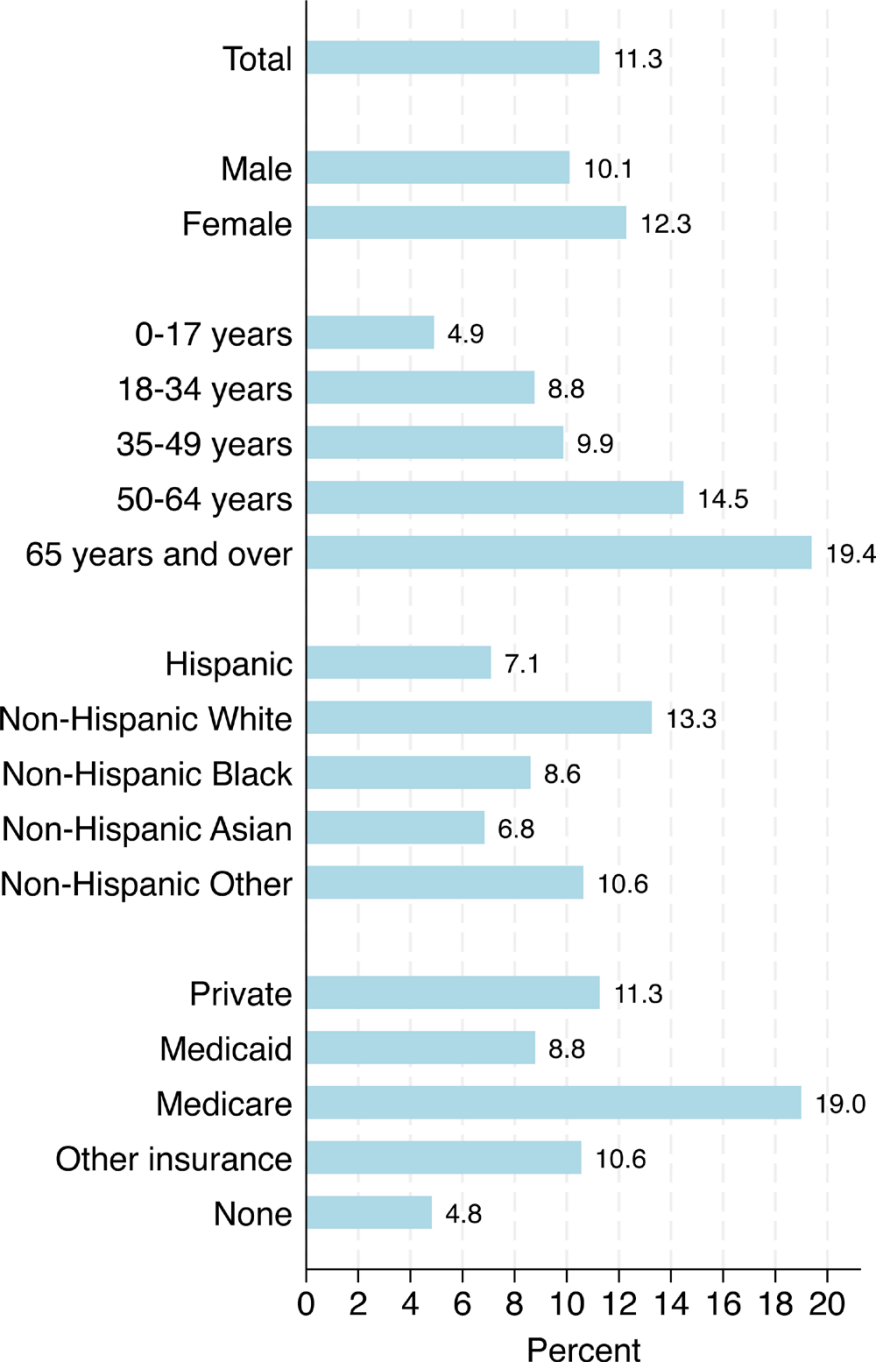
Prevalence of surgical procedures in the United States. Bars signify the weighted reports undergoing surgery based on the proportion of persons reporting at least one surgical procedure. Measures for surgical procedures were analyzed from the National Health Interview Survey 2018 data. Respondents were noninstitutionalized adults in the United States who were asked, “During the past 12 months, (have you/has sample child) had surgery or other surgical procedures either as an inpatient or outpatient?” The non-Hispanic other population includes Native American non-Hispanic and respondents indicating multiple races that did not include Hispanic. Health insurance categories of other health insurance included military health insurance while none included persons with no or unknown health insurance status.

Overall, 11.3% (95% CI = 10.8%–11.7%) of persons reported having surgery in the past year (Fig. 1). Among subgroups, characteristics associated with higher proportions of having surgery in the past year included being female (12.3%, 95% CI = 11.7%–12.9%), age 65 years and over (19.4%, 95% CI = 18.3%–20.4%), non-Hispanic White race (13.3%, 95% CI = 12.7%–13.8%), and Medicare insurance (19.0, 95% CI = 17.7%–20.2%).

## DISCUSSION

Among persons living in households in the United States, 1 in 9 persons reported undergoing at least one surgical procedure in the past year. The highest prevalence was identified among persons 65 years and older and among Medicare beneficiaries, with surgical procedures reported by approximately 1 in 5 persons for both groups. These findings provide a more comprehensive view compared to recent but more restrictive samples.^9^

Interpretation of these data should be considered in the setting of certain limitations. It is not possible to know from this survey whether endoscopy, interventional cardiology procedures, or other less invasive procedures were considered by respondents as surgery, which could impact findings. Surgical procedures vary with respect to invasiveness, complexity, and emergent status, and this heterogeneity represents a level of detail that is not captured by the survey. Also, because the NHIS survey discontinued asking questions about surgery, the ability to generate estimates or trends of the proportion of the population having surgery in more recent years is not possible. While the survey design permits national insights about care patterns in the United States, self-reported data may be subject to recall bias with uncertain reliability or response bias, where, for example, response rates may be higher among older respondents who are more likely to undergo surgery or, conversely, healthier respondents who are less likely to undergo surgery. Finally, these findings do not apply to individuals in noninstitutionalized and long-term care settings, which necessitate different sampling strategies.

Our findings highlight how common persons undergo surgery and identify differences based on relevant demographic and other characteristics. These data provide an important perspective on the current prevalence of surgery among individuals in the United States, which can inform resource planning and workforce needs.

## ACKNOWLEDGMENTS

M.C.B. had full access to all of the data in the study and took responsibility for the integrity of the data and the accuracy of the data analysis. Concept and design, drafting of manuscript, and statistical analysis: M.C.B. Administrative, technical, or material support, and supervision: M.C.B. and T.N. Acquisition, analysis, or interpretation of data, critical revision of the manuscript for important intellectual content, obtained funding: All authors.
